# Phosphorylation of TIP3 Aquaporins during *Phaseolus vulgaris* Embryo Development

**DOI:** 10.3390/cells8111362

**Published:** 2019-10-31

**Authors:** Mark J. Daniels, Mark Yeager

**Affiliations:** 1 Department of Molecular Physiology and Biological Physics, University of Virginia School of Medicine, Charlottesville, VA 22908, USA; 2 Department of Medicine, Division of Cardiovascular Medicine, University of Virginia Health System, Charlottesville, VA 22908, USA; 3 Center for Membrane and Cell Physiology, University of Virginia School of Medicine, Charlottesville, VA 22908, USA; 4 Cardiovascular Research Center, University of Virginia School of Medicine, Charlottesville, VA 22908, USA

**Keywords:** aquaporin, membrane protein, *Phaseolus vulgaris*, phosphorylation, plant development

## Abstract

The membrane phosphoproteome in plant seed changes dynamically during embryo development. We examined the patterns of *Phaseolus vulgaris* (common bean) seed membrane protein phosphorylation from the mid-maturation stage until two days after germination. Serine and threonine phosphorylation declined during seed maturation while tyrosine phosphorylation remained relatively constant. We discovered that the aquaporin PvTIP3;1 is the primary seed membrane phosphoprotein, and PvTIP3;2 shows a very low level of expression. The level of phosphorylated Ser7 in PvTIP3;1 increased four-fold after seed maturation. Since phosphorylation increases water channel activity, we infer that water transport by PvTIP3;1 is highest in dry and germinating seeds, which would be optimal for seed imbibition. By the use of isoform-specific, polyclonal peptide antibodies, we found that PvTIP3;2 is expressed in a developmental pattern similar to PvTIP3;1. Unexpectedly, PvTIP3;2 is tyrosine phosphorylated following seed maturation, which may suggest a mechanism for the regulation of PvTIP3;2 following seed germination. Analysis of protein secondary structure by circular dichroism spectroscopy indicated that the amino-terminal domain of PvTIP3;1 is generally unstructured, and phosphorylation increases polyproline II (PPII) helical structure. The carboxy-terminal domain also gains PPII character, but in a pH-dependent manner. These structural changes are a first step to understand TIP3 aquaporin regulation.

## 1. Introduction

Mammalian aquaporins are generally in a constitutively open conformation, and regulation is achieved by modulating protein abundance through vesicle-mediated transport of the channels to the plasma membrane. However, a number of plant aquaporins are also regulated directly by pH and/or phosphorylation. In fact, phosphorylation of plant water channels appears to be a widespread mechanism for direct regulation of transmembrane water flux [1]. For example, phosphorylation increases the water channel activity of the *Phaseolus vulgaris* bean seed aquaporin PvTIP3;1 (formerly α-TIP) expressed in *Xenopus laevis* oocytes [2]. The heterologously expressed protein shows weak water channel activity that is greatly increased when the oocytes are treated with kinase activating and phosphatase inhibiting compounds [2]. Plant aquaporins are expressed in different tissues, membranes, and developmental stages [3,4]. PvTIP3;1 is the most abundant integral membrane protein in the *P. vulgaris* protein storage vacuole (PSV) [5], but in *Arabidopsis thaliana* it is also found in the plasma membrane [6]. Ser7 at the amino-terminus is phosphorylated in vivo during seed germination by a calcium-dependent protein kinase (CDPK) [7]. Therefore, it may be inferred that PvTIP3;1 phosphorylation enhances the rehydration of PSVs, which would facilitate the enzymatic breakdown of stored compounds and the release of nutrients [8]. PvTIP3;1 Ser7 is the only residue that can be phosphorylated in vitro by protein kinase A (PKA) [9], indicating that this residue is key in the phosphorylation-dependent regulation of PvTIP3;1. A partial cDNA for the close homolog PvTIP3;2 (formerly β-TIP) was initially isolated from developing seed using the full-length cDNA of PvTIP3;1 [10]. Although the transcript was not detected in later studies of *P. vulgaris* [11], the protein was found to express and co-localize with AtTIP3;1 in *A. thaliana* seed [6]. After germination, the TIP3 aquaporins are degraded, while TIP1 proteins accumulate in the developing seedling [12,13].

Protein phosphorylation in ripening seed and the germinating embryo is a dynamic process and a key regulatory mechanism [14]. Here, we examined how the phosphorylation of *P. vulgaris* membrane proteins changes during seed maturation and germination and how this might correspond to aquaporin phosphorylation. We then used circular dichroism (CD) spectroscopy to examine possible phosphorylation-dependent structural changes of the amino- and carboxy-tail domains of PvTIP3;1.

## 2. Materials and Methods

### 2.1. Chemicals and Reagents

All chemicals and reagents were American Chemical Society Reagent grade or better and obtained from Sigma-Aldrich (St. Louis, MO, USA) unless otherwise noted. Liquid measures are by volume unless indicated otherwise. All solutions were prepared in purified deionized water unless otherwise indicated. Reactions and incubations were performed at room temperature (~22 °C) unless otherwise described.

### 2.2. Peptide Synthesis and Purification

Peptides ATTRYSFGRTDEAC (aTIPnt13C), ATRRYSFGRTDEAT (aTIPnt14), ATRRY(pS)FGRTDEAT (aTIPnt14_pS7), ATTRY(pS)FGRTDEAC (aTIPnt13C_pS7), ATTRRYEFGMNEASHC (bTIPnt15C) and YEYAVIPIEPPPHHHQPLATEDY (aTIPct23), were synthesized and purified by the Microprotein Sequencing and Peptide Synthesis Facility at the University of North Carolina at Chapel Hill. All peptides were prepared with an acetylated amino-terminus and an amidated carboxy-terminus except for aTIPct23, which was prepared with a free acid at the carboxy-terminus. The carboxy-terminal cysteines of aTIPnt13C, aTIPnt13C_pS7, and bTIPnt15C were added to facilitate subsequent conjugation reactions.

### 2.3. Membrane Protein Isolation

Membranes were isolated from seeds of the common bean (*Phaseolus vulgaris* L. cv. Blue Lake Bush 274) as described previously [15], with modifications. Fresh green immature and yellowing mature seeds were obtained from local markets (San Diego, CA, USA). Dry seeds were obtained from W. Atlee Burpee and Company (Warminster, PA, USA). Germination of dry seeds was performed by imbibition on a thin layer of tap water for 1–2 days.

Membranes were isolated at 4 °C unless otherwise noted. Germinated or immature seeds were homogenized in a mini-blender with Buffer A (50 mM triethanolamine (TEA) (pH 8.0), 0.5% polyvinylpyrrolidone, 5 mM EDTA, 5 mM EGTA, 5 mM benzamidine, 5 mM naphthyl acid phosphate, 2.7 mM Na_3_VO_4_, 2 mM butylated hydroxytoluene, 1 mM 1,10-phenanthroline, 200 nM okadaic acid). Dry seeds were first pulverized with an electric coffee mill (Braun, “espresso” setting, Kronberg, Germany) and then homogenized in Buffer A using a tissue homogenizer (Tekmar, Cincinnati, OH, USA). The homogenate was filtered through cheesecloth, and starch granules were removed by centrifugation for 10 min at 800× *g*. The remaining microsomes were removed from the supernatant by centrifugation for 60 min at 25,000× *g*. The microsomal pellet was resuspended by sonication in Buffer C (25 mM TEA (pH 7.5), 1 mM EDTA, 1 mM EGTA, 1 mM benzamidine, 3 mM β-mercaptoethanol) and pelleted by ultracentrifugation for 60 min at 60,000× *g*. Washed microsomes were resuspended by sonication in Buffer B (1 M potassium iodide, 50 mM TEA (pH 7.5), 10 mM Na_2_S_2_O_3_, 5 mM benzamidine), incubated on ice for 30 min, and pelleted as before. Membranes were resuspended by sonication in Buffer C and collected on a cushion of 60% sucrose in Buffer C by ultracentrifugation for 60 min at 25,000× *g*. The membranes were resuspended in Buffer C, pelleted by ultracentrifugation as before, and resuspended in a minimal volume of Buffer D (10 mM TEA (pH 7.5), 20% glycerol, 1 mM EDTA). Isolated membranes were stored at –20 °C. Protein concentration was determined using a bicinchoninic acid assay (Pierce Biotechnology Rockford, IL, USA).

Lipids were removed from membrane protein fractions by solvent extraction. An aqueous membrane suspension was diluted with four volumes of methanol, one volume of chloroform, and three volumes of water. The aqueous and organic fluid phases were vortex-mixed and then separated by centrifugation for five min at 13,000× *g*. The upper organic phase was discarded, and the interface and lower aqueous phase were diluted with five volumes of methanol. Precipitated proteins were isolated by centrifugation for five min at 13,000× *g*. The supernatant was discarded, and the protein pellet was resuspended and washed with five volumes of acetone. Precipitated proteins were again isolated by centrifugation as before. The supernatant was discarded, and the protein pellet was air-dried. A one-third volume of 10% sodium dodecyl sulfate (SDS) was added to the pellet, which was then allowed to dissolve undisturbed for 12 h. The dissolved proteins were diluted to its original volume with water, and then a 1/10 volume of 1 M dithiothreitol (DTT) was added. One volume of 20% glycerol, 50 mM Tris-HCl (pH 6.8) in water was then added, and the solution was incubated for 20 min at 50 °C.

Protein concentrations were determined using a bicinchoninic acid assay (Pierce BCA Protein Assay Kit, Pierce Biotechnology). SDS polyacrylamide gel electrophoresis (SDS-PAGE) chromatography was performed with ~10 μg aliquots of protein per lane using 4–20% linear acrylamide gradient Tris-glycine gels (Novex/Invitrogen, Carlsbad, CA, USA). PeppermintStick phosphoprotein markers (Molecular Probes, Eugene, OR, USA) and broad range prestained protein markers (New England Biolabs, Ipswitch, MA, USA) were used as molecular weight standards. Electroblotting for Western analysis was performed as previously described [16], using pure nitrocellulose membranes (Protran BA-85) and modified Towbin’s buffer (25 mM Tris base, 192 mM glycine, 20% methanol, 0.05% SDS). Digital images of SDS-PAGE gels and immunoblots were recorded using an optical scanner (model 5370c, Hewlett-Packard, Palo Alto, CA, USA). Images were then converted to greyscale, and brightness and contrast were optimized using Photoshop (Adobe, San Jose, CA, USA). Correlating the molecular weight of reference proteins to their migration distance in SDS-PAGE yielded a distance-to-weight conversion that was used to calculate protein molecular weight.

### 2.4. Phosphoprotein Detection

Phosphoprotein staining was performed using the Pro-Q Diamond stain (Invitrogen, Eugene, OR, USA), following the manufacturer’s instructions. The Pro-Q Diamond fluorescent dye is highly specific for phosphorylated serine, threonine, and tyrosine residues on peptides and proteins, and will bind these residues in a sequence-independent manner [17]. Stained gels were viewed and digitized using an Eagle Eye II gel documentation system (Stratagene, San Diego, CA, USA).

### 2.5. Phosphoamino Acid Detection

Western immunoblots for phosphoproteins were blocked with 5% IgG-free bovine serum albumin (BSA) in TBS (25 mM Tris-HCl (pH 7.4), 137 mM NaCl). Phosphoserine (1), phosphothreonine (2), and phosphotyrosine (3) immunoblot analyses were performed with (1) antisera PSR-45 (Sigma-Aldrich) diluted 1/800 with 3% IgG-free BSA in TBST (25 mM Tris-HCl (pH 7.4), 137 mM NaCl, 0.2% Tween-20), (2) antisera PT-5H5 (Zymed Laboratories, San Francisco, CA, USA) diluted 1/500 with 3% IgG-free BSA in TBST, and (3) antisera PT-66 (Sigma-Aldrich) diluted 1/30,000 with 3% IgG-free BSA in TBST, respectively. Antibody solutions were applied for four hours, and the nitrocellulose membranes were then washed four times for 15 min with TBST and incubated for one hour with goat anti-mouse IgG antisera conjugated to horseradish peroxidase (BioRad, Hercules, CA, USA) diluted 1/60,000 with 3% IgG-free BSA in TBST. The immunoblots were then washed five times for 15 min with TBST and once for 15 min with TBS. Antibody labeling was detected using the SuperSignal West Pico chemiluminescent substrate (Pierce Biotechnology, Rockford, IL, USA) and recorded onto X-ray film (Kodak, Rochester, NY, USA). The films were digitized, and quantification of protein immunolabeling was performed using the gel analysis routines in the ImageJ software package. To reprobe the immunoblots, nitrocellulose membranes were stripped by washing with 100 mM glycine (pH 2.5) for 2 h at 37 °C, followed by two rinses with TBS and two rinses with TBST. To further remove bound antibodies, each membrane was then incubated for 23 h in TBST supplemented with 0.1% of the free phosphoamino acid, corresponding to the antisera used on the membrane.

### 2.6. PvTIP3;1 and PvTIP3;2 Immunodetection

Immunolabeling using polyclonal antibodies to full-length PvTIP3;1 [5] was performed by a four hr incubation with the antisera diluted by 1/30,000 with 1% gelatin and 0.1% PVP in TBST. The nitrocellulose membrane was then washed four times for 15 min with TBST and incubated for one hr with goat anti-rabbit IgG antisera conjugated to horseradish peroxidase (BioRad) diluted 1/50,000 in 1% gelatin, 0.1% PVP in TBST. The immunoblot was then washed five times for 15 min with TBST and once for 15 min with TBS. Antibody labeling was detected using the SuperSignal West Pico chemiluminescent substrate (Pierce Biotechnology) and recorded onto X-ray film (Kodak).

Peptide aTIPnt13C and aTIPnt13C_pS7 antisera were prepared by Pacific Immunology (Ramona, CA, USA). The peptides were conjugated to keyhole limpet hemocyanin (KLH) and injected into New Zealand White rabbits, with booster injections at three- to four-week intervals. Complete Freund’s adjuvant was used for the first immunization, and incomplete Freund’s adjuvant was used for subsequent immunizations. Highly specific antisera were obtained after two boosts, and sera after the fourth boosts were used for immunoblot analysis. Peptide bTIPnt15C antisera were prepared by Pocono Rabbit Farm and Lab (Canadensis, PA, USA). This peptide was conjugated to KLH and injected into guinea pigs, with booster injections at two- to four-week intervals. Complete Freund’s adjuvant was used for the first immunization and incomplete Freund’s adjuvant was used for subsequent immunizations. Highly specific antisera were obtained after the second boost, and sera after the fourth boost were used for immunoblot analysis. Western immunoblots were blocked with Odyssey Blocking Buffer (OB Buffer; Li-Cor, Lincoln, NE, USA) and then immunolabeled four hrs with the peptide antisera diluted by 1/20,000 in the same buffer. For some experiments the bTIPnt15C antiserum was incubated with the PvTIPnt14 peptide at 0.2 mg/mL in 100 μL OB Buffer for two hrs prior to final dilution in OB Buffer and immunolabeling. The nitrocellulose membrane was then washed three times for 15 min with TBST and incubated for one hr with either goat anti-rabbit IgG or donkey anti-guinea pig IgG antisera conjugated to either IRDye680 or IRDye800 (Li-Cor) diluted 1/25,000 in OB Buffer. The immunoblot was then washed five times for 15 min with TBST and once for 15 min with TBS. Antibody labeling was detected using an infrared scanner (Odyssey Infrared Imager, Li-Cor). Linear adjustments to the brightness and contrast of the entire blot image were made in Photoshop (Adobe) to remove background noise. Gel staining and blot labeling intensities were quantified from digitized images using the NIH-ImageJ software package (http://rsb.info.nih.gov/ij/, National Institutes of Health, Bethesda, MD, USA). PvTIP3;1 treated with either the catalytic subunit of protein kinase A or with λ-protein phosphatase (New England BioLabs) served as positive and negative controls, respectively, for protein phosphorylation [9]. A phosphoprotein mix (PeppermintStick Phosphoprotein Molecular Weight Standards, Invitrogen) was used as a positive control for serine phosphorylation, and both rabbit polyclonal poly-Z-PS1 (Invitrogen) and mouse monoclonal pSer-7F11 (Invitrogen) were used as alternate phosphoserine antisera in addition to PSR-45.

### 2.7. Overexpression of PvTIP3;2 in P. pastoris

A synthetic gene corresponding to the complete amino acid sequence of PvTIP3;2 (Genbank EST GW888595) but with a *P. pastoris* codon bias was generated by GenScript (Piscataway, NJ, USA). Cloning, transformation, and protein overexpression in *P. pastoris* strain KM71H were performed as previously described [9].

### 2.8. Phosphotyrosine Immunoprecipitation

Aliquots of detergent-solubilized membrane proteins (25 μg) were diluted to a volume of 490 μL in 40 mM Tris-HCl (pH 6.8), 0.025% Triton X-100, and 1.4 mM ß-mercaptoethanol. Since the PT-66 monoclonal antibody specifically immunolabels and immunoprecipitates phosphotyrosinylated proteins [18,19], an aliquot (20 μL) of the antibody coupled to agarose beads (Sigma-Aldrich) was added to the sample and incubated with gentle mixing for 17 h at 4 °C. The antibodies were pelleted by centrifugation at 2000× *g* for 30 s, and the supernatant was discarded. The resin was resuspended in 1.5 mL TBST, incubated with gentle mixing for 20 min, and pelleted as before. This washing step was repeated three times. The washed resin pellet was then resuspended in 30 μL Denaturing Buffer (4% SDS, 20% glycerol, 100 mM Tris-HCl (pH 6.8), 100 mM DTT), incubated at 70 °C for 30 min, and then pelleted as before. The supernatant was evaluated by SDS-PAGE chromatography, Western immunoblotting, and either TIP3;1 or TIP3;2 immunodetection as described above.

### 2.9. Mass Spectrometry

SDS-PAGE gel bands of membrane proteins from seed imbibed for 24 h that showed intense labeling with the Pro-Q Diamond Phosphoprotein stain (Molecular Probes, Inc., Eugene, OR, USA) were excised using an ethanol-washed razor blade. Each ~5 × 1 mm gel slice was then diced into ~1 mm^3^ cubes, which were transferred to a siliconized 1.5 mL polypropylene tube. In-gel trypsin digestion and matrix-assisted laser desorption/ionization-time of flight (MALDI-TOF) mass spectrometry (MS) of the released peptides was performed as previously described [9].

### 2.10. Circular Dichroism (CD) Spectrometry

Circular dichroism (CD) spectra were recorded using AVIV model 202 and model 410 spectropolarimeters controlled by the CDS software package (Aviv Biomedical, Lakewood, NJ, USA). Spectrometer performance was calibrated as previously described [20], using (+)-camphor-10-sulfonic acid to check CD signal gain and holmium oxide to verify wavelength accuracy. Peptides were completely soluble in aqueous solution, and stock solutions of 2 to 10 mM in water were prepared just before use. The absence of significant aggregation was verified by optical absorbance scanning of the peptide solutions. Peptide concentrations were determined by measuring the solution absorbance at 280 nm and using the extinction coefficient of tyrosine in water (1490 M^−1^cm^−1^ at 280 nm [21]). Peptide stock solutions were diluted to 87 μM in buffer just before use. To obtain a buffer pH of 5.0, peptide stock solutions were diluted into 5 mM sodium acetate buffer, and for a pH of 6.0, 7.0, or 8.0, peptides were diluted into 5 mM of the appropriate sodium phosphate buffer [16]. For data collection, a strain-free quartz cuvette of 0.10 cm path length was used, and sample temperatures were held at 5.0, 15.0, or 25.0 °C. Spectra were recorded with a 1.0 nm bandwidth and a 0.5 nm step-size over the wavelength range of 180 to 270 nm, with an averaging time of 10 sec per step. By use of these parameters, the spectropolarimeter photomultiplier tube dynode voltage never exceeded 500 VDC while recording spectra, thus maintaining a good signal-to-noise ratio [22]. Three scans were averaged for both sample and buffer in order to improve the signal-to-noise ratio. The CDtool software package [23] was used for processing the collected data (version 1.4, Birkbeck College, London, England). Final spectra were obtained by subtracting buffer spectra from the sample spectra, and CD intensities were then set to an average of zero in the range of 250 to 260 nm, where polypeptides exhibit minimal CD signal [24,25]. The online resource Dichroweb [26] was used to analyze the CD results. Spectral deconvolution to calculate secondary structure solutions was performed using the CDSSTR method [25], which is based on a reference dataset of 128 membrane and soluble protein structures [27].

## 3. Results

Membrane fractions were isolated from seeds of *P. vulgaris* at five stages of development: (1) greening immature seed, (2) green mature seed, (3) dry seed, (4) seed imbibed for 24 h, and (5) seed imbibed for 48 h. Vanadate was used as a broad-spectrum kinase and phosphatase inhibitor [28]. Samples were delipidated thoroughly by solvent extraction with chloroform and methanol to prevent interference from contaminating phospholipids.

### 3.1. Developmental Changes in the Abundance of Seed-Membrane Phosphoproteins

**Figure 1:**
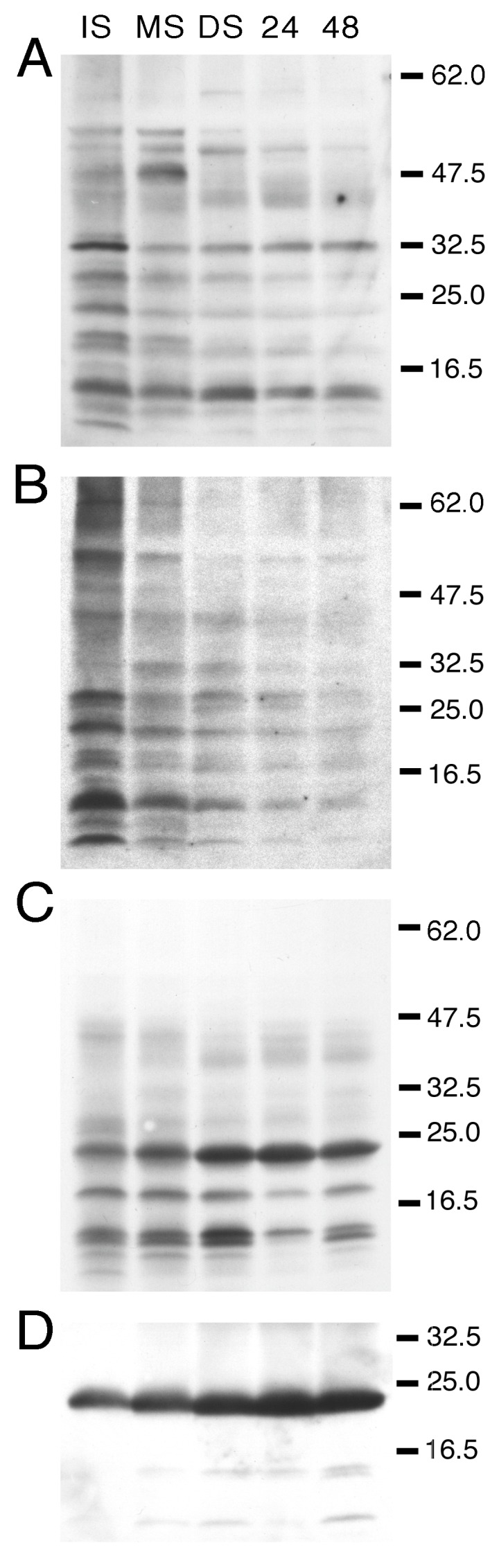
Western immunoblots probed either with (**A**) phosphoserine, (**B**) phosphothreonine, or (**C**) phosphotyrosine antisera. Blot lanes: IS, immature seed; MS, mature seed; DS, dry seed; 24, seed imbibed for 24 h; 48, seed imbibed for 48 h. Blot from panel **C** stripped and reprobed with TIP3 antisera shown in panel **D**. Position of molecular weight markers (kDa) indicated on right.

To examine membrane phosphoproteins in *P. vulgaris* seeds, the membrane protein fractions were separated by SDS-PAGE and examined by Western immunoblot analysis using phosphoamino acid-specific antisera. Each immunoblot was then stripped and reprobed with antisera to full-length PvTIP3;1 in order to correlate the developmental changes in phosphorylation with PvTIP3 abundance. A variety of membrane proteins displayed serine phosphorylation (Figure 1A), which was generally highest in immature seed and declined at later developmental stages. Labeling of 34, 12, and 11 kDa proteins remained relatively constant (Figure S1). One small protein of 5 kDa was labeled in immature seed but not at later stages. A 46 kDa protein showed weak labeling in immature seed, strong labeling in yellowing mature seed, and no labeling thereafter. One protein of 50 kDa showed maximal phosphoserine labeling in dry seed membranes and was weakly labeled in all other developmental stages. A number of proteins showed threonine phosphorylation (Figure 1B), again with the general trend that labeling was most intense for proteins in immature seed and either became significantly less or disappeared entirely at later developmental stages. One exception to this was a protein of 34 kDa that was primarily labeled in mature and dry seed. Compared with serine and threonine phosphorylation, fewer membrane proteins were tyrosine-phosphorylated (Figure 1C). In this case labeling remained fairly constant during seed development and germination. Maximal labeling of a 24 kDa protein occurred in dry and imbibed seed, with less labeling in mature seed and even weaker labeling in immature seed. Proteins of 18 and 14 kDa were labeled at all stages but showed reduced labeling in 24 h imbibed seed. No labeling was seen when a duplicate immunoblot was treated with λ-protein phosphatase prior to immunolabeling with phosphotyrosine antibodies (data not shown), demonstrating that the labeling was phosphospecific. When the immunoblot was stripped and reprobed with antisera to full-length PvTIP3;1, a ~24 kDa protein was intensely labeled, especially in dry seed and seed imbibed for 24 h (Figure 1D).

**Figure 2:**
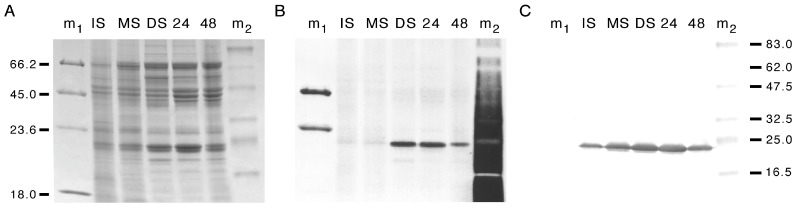
Developmental changes in the abundance of *P. vulgaris* seed membrane proteins showing that PvTIP3 proteins are the primary phosphoproteins. (**A**) Coomassie and (**B**) phosphoprotein stained gels. (**C**) Immunoblot using antisera to PvTIP3 proteins. Gel lanes: IS, immature seed; MS, mature seed; DS, dry seed; 24, seed imbibed for 24 h; 48, seed imbibed for 48 h. Lane m1, Peppermint Stick phosphoprotein markers; lane m2, broad-range prestained molecular weight standards. Position of Peppermint Stick markers indicated at left along with the respective molecular weight in kDa. Position of broad-range prestained molecular weight standards (kDa) indicated at right. Background fluorescence in lane m2, panel **B** is from phenol red used by the manufacturer in the marker protein solution.

A Coomassie-stained SDS-PAGE gel of the same membrane protein fractions showed developmental changes in the abundance of several major and minor membrane proteins (Figure 2A, Table 1) that resembled the staining patterns seen previously with membrane preparations from *P. vulgaris* seeds [5]. Pro-Q Diamond phosphoprotein staining showed a single major membrane phosphoprotein band of 24 kDa that appeared at the dry seed stage (Figure 2B, Table 2) for which the staining intensity then decreased over the two days following imbibition. A minor phosphoprotein of 19 kDa was only observed in dry seed membranes.

### 3.2. PvTIP3 Proteins Are the Primary Seed-Membrane Phosphoproteins

A protein band of ~23 kDa was immunolabeled by antisera to full-length PvTIP3;1 (Figure 2C), which showed a similar pattern of expression during seed maturation and germination as the 24 and 22 kDa proteins detected by Coomassie staining (Figure 2A). Previous results have shown that PvTIP3;1 typically migrates as a protein of 25 to 30 kD in SDS-PAGE [5,15], along with an antigenically related protein of 23 kDa that appears to be a processed form of the protein [5]. The small variations in molecular weight may be due to differences in chromatographic methods or the result of membrane protein delipidation. We note that the antisera used in this experiment may also label the tonoplast aquaporin TIP2;1 [29] (formerly termed δ-TIP [30]). However, previous observations indicate that TIP2;1 is not present in seeds of *A. thaliana* [6,31], although in *Hevea vulgaris* (barley) *HvTIP2;1* does show significant RNA expression in mature seed [32].

**Figure 3:**
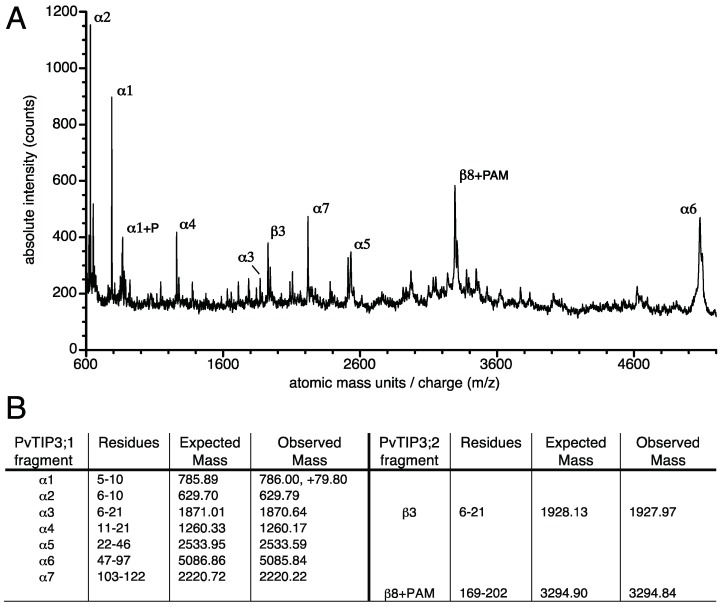
(**A**) MALDI-TOF MS of the trypsin-digested primary phosphoprotein from membranes of dry seed. (**B**) Mass predicted and observed for tryptic fragments of PvTIP3;1 and PvTIP3;2. P indicates phosphorylated peptide, PAM denotes acrylamide adduct. Expected mass list presented as the expected average protonated mass. Mass accuracy using external standards was ≤0.02%.

To confirm that PvTIP3 proteins are the major phosphoproteins of bean seed membranes, the SDS-PAGE gel band that was strongly labeled by the Pro-Q Diamond phosphoprotein stain (Figure 2B) was excised and treated with trypsin. The peptides released by this proteolytic digestion were then characterized by MALDI-TOF MS. Several peptides corresponding to PvTIP3;1 were observed, including one showing phosphorylation of Ser7. Two additional fragments were also detected (Figure 3), which correspond to PvTIP3;2, one of which was interpreted as a peptide with an acrylamide moiety adducted to Cys197 of PvTIP3;2. Such a protein modification is not uncommon on cysteine residues when proteins are purified by SDS-PAGE [33]. Nevertheless, the two putative peptides of PvTIP3;2 are insufficient to unequivocally identify this protein, which has not been previously observed in conjunction with PvTIP3;1 [9]. AtTIP3;2 has been observed along with AtTIP3;1 in the seed of *A. thaliana*, however [6]. In addition, no mass peaks corresponding to PvTIP2;1 (GenBank ID AAZ78660) were observed in tryptic digests of either the excised phosphoprotein or of purified PvTIP3;1 (data not shown). The absence of any other significant peptides indicates that PvTIP3;1 is the primary membrane phosphoprotein in *P. vulgaris* seed development.

### 3.3. Expression of PvTIP3;1 and PvTIP3;2 in Seed Development

To verify that our aTIPnt14 and aTIPnt14_pS7 antisera were specific for PvTIP3;1, we overexpressed PvTIP3;1 and PvTIP3;2 in *Pichia pastoris* and performed immuno-dot blot analysis (Figure S2). Indeed there was no cross-reactivity of these antibodies with PvTIP3;2 using the same conditions that resulted in labeling of PvTIP3;1. However, we observed that the original PvTIP3;1 antiserum generated against full-length protein [5] also labeled PvTIP3;2, which is not surprising given the 75% identity between the two proteins (Figure S1). This indicates that the labeling in Figure 1D corresponds to both PvTIP3;1 and presumably a small quantity of PvTIP3;2.

**Figure 4:**
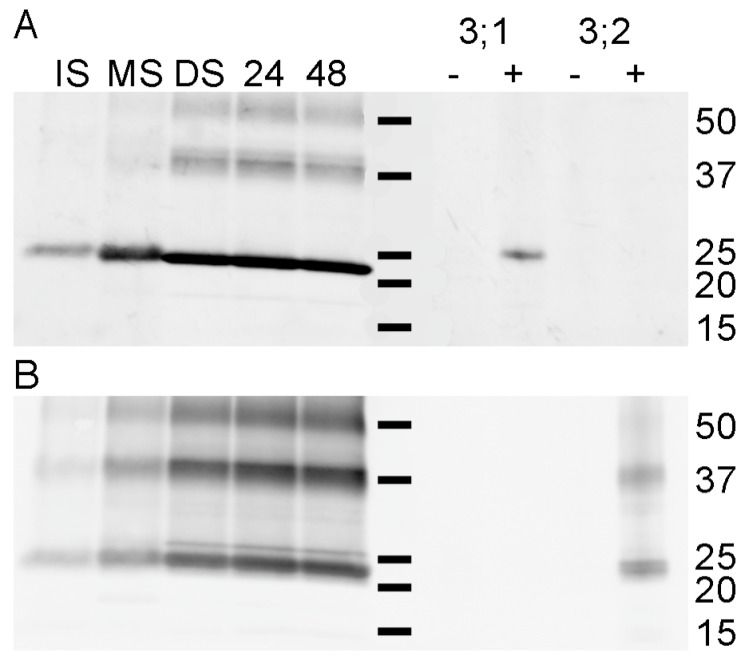
Immunodetection of PvTIP3;1 and PvTIP3;2 in seed membranes. Western immunoblots colabeled with (**A**) aTIPnt13C and (**B**) bTIPnt15C antisera. Blot lanes: IS, immature seed; MS, mature seed; DS, dry seed; 24, seed imbibed for 24 h; 48, seed imbibed for 48 h. Proteins of 40 and 57 kDa were also labeled and are likely oligomers as observed previously with PvTIP3;1 [5,15,34]. To monitor antisera specificity, labeling controls for PvTIP3;1 (**3;1**) and PvTIP3;2 (**3;2**) shown on right, with aliquots from washed microsomes of PICZ-vector only transformed *Pichia pastoris* (**-**) or yeast expressing the indicated protein (**+**). Position of molecular weight markers (central bars) with kDa values at the right.

In order to distinguish the developmental expression patterns of the two TIP3 proteins, we generated antisera to PvTIP3;2 using a peptide with the least identity to PvTIP3;1 as the antigen (Figure S1). Producing these amino-terminal antibodies (designated antisera bTIPnt15) in guinea pigs rather than rabbits provided us with the ability to perform co-labeling experiments using both aTIPnt14 and bTIPnt15 antibodies. We therefore immunolabeled membrane protein fractions with antisera specific for each isoform. Proteins of ~24 kDa were immunolabeled by aTIPnt14 and bTIPnt15 antisera (Figure 4), both showing a pattern of labeling that recapitulated the results with antisera to full-length PvTIP3;1 (Figure 2C); that is, phosphoproteins accumulated through seed maturation and reached a plateau from the dry seed stage through germination.

### 3.4. Ser7 Phosphorylation in PvTIP3;1 Increases Following Seed Maturation

**Figure 5:**
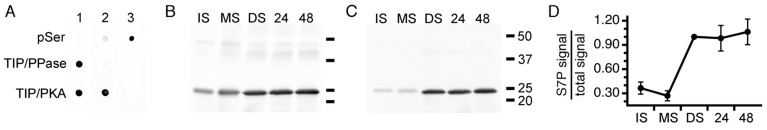
Immunodetection of PvTIP3;1 phosphorylated at Ser7. (**A**) Dot blots of a phosphoserine-bearing protein mix (pSer) and of purified PvTIP3;1 treated with either lambda protein phosphatase (TIP/PPase) or protein kinase A catalytic subunit (TIP/PKA). Blots were immunolabeled with aTIPnt13C (lane 1), aTIPnt13C_S7P (lane 2), or phosphoserine (lane 3) antisera to test antibody specificity. Western immunoblots using (**B**) aTIPnt13C or (**C**) aTIPnt13C_S7P antisera. Blot lanes: IS, immature seed; MS, mature seed; DS, dry seed; 24, seed imbibed for 24 h; 48, seed imbibed for 48 h. Position of molecular weight markers (bars) with kDa values indicated to the right of panel **C**. (**D**) Graph showing ratio of aTIPnt13C_S7P to aTIPnt13C antisera labeling. Results displayed as the average of four experiments ± one standard deviation of the sample, normalized so that the labeling ratio is 1:1 for the dry seed fraction. Horizontal axis indicates seed fractions as in **B** and **C**.

Previous studies have shown that Ser7 of PvTIP3;1 is phosphorylated in vivo [7], and we demonstrated that Ser7 is in fact the only serine residue that can be phosphorylated when PvTIP3;1 is expressed in *P. pastoris* yeast [9]. To study the changes in phosphorylation of Ser7 during seed development, antibodies were generated against the unphosphorylated amino-terminal peptide (designated antisera aTIPnt14) as well as the Ser7-phosphorylated amino-terminal peptide (designated antisera aTIPnt14_pS7). Antibodies against aTIPnt14_pS7 were highly specific for phosphorylated PvTIP3;1, whereas the aTIPnt14 antiserum labeled both phosphorylated and dephosphorylated forms (Figure 5A). Curiously, a commercial phosphoserine antiserum (PSR-45) did not label Ser7-phosphorylated PvTIP3;1, even under conditions in which it labeled a mix of proteins containing phosphoserine (Figure 5A). Alternate rabbit polyclonal (poly-Z-PS1) and mouse monoclonal (pSer-7F11) phosphoserine antisera also showed no labeling of PKA-phosphorylated PvTIP3;1 in conditions that labeled control phosphoproteins (data not shown). Immunolabeling using the polyclonal peptide antibodies (Figure 5B,C) was similar to the developmental changes in phosphorylation that were detected using the original polyclonal antisera raised against full-length PvTIP3;1 [5]. When compared to the labeling of total protein, phosphorylation of Ser7 was lowest in developing seed, followed by a four-fold increase in dry seed, which remained constant during maturation (Figure 5D). We note that our results in Figure 1A do not show this substantial increase in phosphorylation, consistent with our observation that phosphorylated PvTIP3;1 is not labeled by phosphoserine antisera (Figure 5A).

### 3.5. Tyrosine Phosphorylation of the PvTIP3;2 Amino-Terminal Peptide

**Figure 6:**
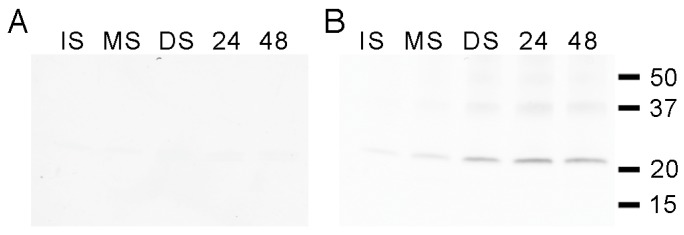
Seed membrane fractions immunoprecipitated with phosphotyrosine antisera, then fractionated by SDS-PAGE and co-labeled with either (**A**) aTIPnt13C antisera against PvTIP3;1 or (**B**) bTIPnt15C antisera against PvTIP3;2. Blot lanes: IS, immature seed; MS, mature seed; DS, dry seed; 24, seed imbibed for 24 h; 48, seed imbibed for 48 h. Position of molecular weight markers (kDa) indicated on right.

The co-migration of a phosphotyrosinylated protein with the two PvTIP3 proteins (Figure 1C,D) suggested that one or both are tyrosine phosphorylated. In order to identify the phosphotyrosinylated protein, seed membrane proteins were immunoprecipitated using an anti-phosphotyrosine antibody coupled to agarose beads. The immunoprecipitated proteins were then separated by SDS-PAGE, electroblotted to a nitrocellulose membrane and co-immunolabeled with TIP3;1 and TIP3;2 antisera (Figure 6). A protein of ~24 kDa was labeled by the TIP3;2 antisera, primarily in fractions derived from dry, 24 h- and 48 h-imbibed seed (Figure 6B), indicating that PvTIP3;2 is tyrosine phosphorylated. Proteins of 40 and 57 kDa were also labeled and are likely oligomers of PvTIP3;2.

### 3.6. Structural Changes in the PvTIP3;1 Amino Terminal Peptide upon Phosphorylation

Phosphorylation of Ser7 in PvTIP3;1 by a calcium-dependent protein kinase has been observed in vivo [7,9], which was associated with an increase in water permeability when this aquaporin was expressed in *Xenopus* oocytes [2]. This is curious since aquaporin phosphorylation typically occurs on residues in extracellular loops and the carboxy terminus [35]. The amino-terminus of these aquaproteins often encodes the protein kinase A-targeting motifs RRxS/T and others [36], but amino-terminal phosphorylation has so far only been observed with PvTIP3;1.

**Figure 7:**
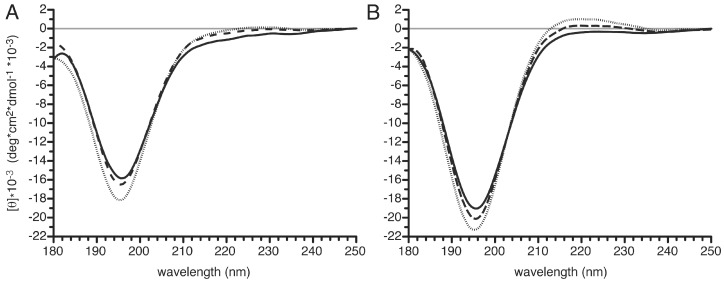
Circular dichroism (CD) spectroscopy of *P. vulgaris* TIP3;1 amino termini. CD spectra were recorded with peptides at 87 μM in buffer of pH 7.0 in 5 mM sodium phosphate. Samples were maintained at 5, 15, or 25 °C (dotted, dashed, or solid line, respectively). CD signal shown in units of molar ellipticity [θ] (deg cm^2^/dmol). (**A**) CD spectra of unphosphorylated peptide aTIPnt14 exhibited strong negative ellipticity (−15,800 deg cm^2^ dmol^−1^) at 195 nm at pH 8 that intensified to −18,200 deg cm^2^ dmol^−1^ as the temperature was lowered. (**B**) CD spectra of phosphorylated peptide aTIPnt14_pS7 showed more negative ellipticity (−18,500 deg cm^2^ dmol^−1^) at 196 nm that intensified to −21,100 deg cm^2^ dmol^−1^ as the temperature was lowered. A broad, weak CD band at ~220 nm (ellipticity ~1000 deg cm^2^ dmol^−1^) also appeared as the temperature was lowered.

Phosphorylation is known to have distinct effects on protein structure [37], and therefore we used CD spectroscopy to explore possible conformational changes upon Ser7 phosphorylation of an amino-terminal peptide. Since PvTIP3;1 is amino-terminally processed in vivo [9] the peptides were prepared accordingly, without the initial methionine and with an acetylated amino terminus. To observe any pH-dependent structural changes, spectra were collected over a pH range of 5 to 8, and at pH 8 with the sample cooled to either 15.0 or 5.0 °C. CD spectra of unphosphorylated (aTIPnt14) and phosphorylated (aTIPnt14_pS7) peptides (Figure 7A,B, respectively) showed strong negative ellipticity at 195–196 nm, which became more negative as the temperature was lowered from 25.0 to 5.0 °C. The phosphorylated peptide also showed a broad, weak CD signal at ~220 nm that appeared as the temperature was lowered (Figure 7B). No differences in the CD spectra were observed over the range of sample pH examined (data not shown). Spectral deconvolution of aTIPnt14 using CDSSTR [25] indicated that the unphosphorylated peptide was fairly unstructured, averaging 2% α-helix, 36% β-strand, 16% β-turn, and 49% unordered structure (Table S2). Similar analysis of aTIPnt14_pS7 indicated that the phosphorylated peptide was also unstructured, with 0% α-helix, 36% β-strand, 12% β-turn, and 51% unordered structure (Table S2). A strong negative signal at 195 nm in conjunction with a weak positive band at 215–220 nm is a hallmark of polyproline II (PPII) helical structure structure [38]. The magnitude of these two bands increases with decreasing temperature, which is another characteristic of PPII structure [39], suggesting a concomitant increase in PPII helical content. Our data do not extend sufficiently far into ultraviolet (UV) wavelengths to quantitate PPII helical content. In addition, the CD spectrum of a random coil also shows a minimum between 190 and 198 nm [40] confounding the analysis of PPII content.

### 3.7. pH- and Temperature-Dependent Structural Changes in the Carboxy Terminus of PvTIP3;1

**Figure 8:**
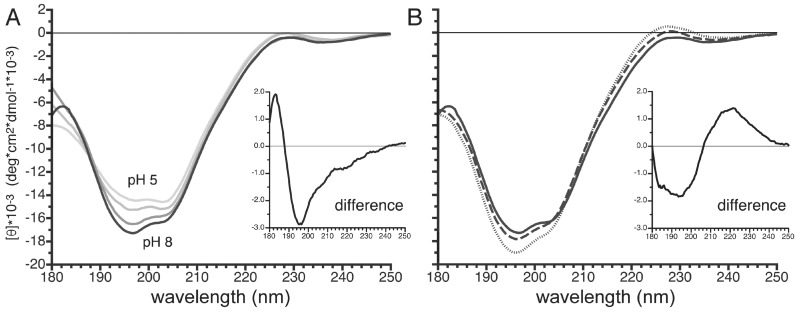
CD spectroscopy of the *P. vulgaris* TIP3;1 carboxy terminal domain. (**A**) Spectra collected with peptides at 50 μM in buffer of 5mM sodium acetate pH 5.0 or in buffer of 5 mM sodium phosphate pH 6.0, 7.0, or 8.0 (line darkens with increasing pH) with sample at 25 °C. Inset shows difference between pH 5 and pH 8 spectra. (**B**) Spectra collected at pH 8.0, at a temperature of 5, 15, or 25 °C (dotted, dashed, or solid line, respectively). Inset shows difference between 25 °C and 5 °C spectra. CD signal shown in units of molar ellipticity [θ] (deg cm^2^/dmol). At pH 5 two strong negative peaks were observed at 203 nm (ellipticity −14,600 deg cm^2^ dmol^−1^) and 197 nm (ellipticity −14,500 deg cm^2^ dmol^−1^). As the pH was shifted to 8, the band at 197 nm became dominant, reaching a maximum of −17,300 deg cm^2^ dmol^−1^.

CD spectra were also recorded for a peptide corresponding to the 23 carboxy-terminal amino acids of PvTIP3;1 (designated aTIPct23). At higher wavelengths spectra showed a weak minimum at ~235 nm and a weak maximum at ~228 nm (Figure 8A). At lower wavelengths spectra displayed strong negative bands at 197 and 203 nm. As the pH increased from 5–8, the two strong minima increased unevenly such that at pH 8.0 the band at 203 nm appeared as a shoulder of the primary minimum at 197 nm. Spectral deconvolution using CDSSTR indicated that the peptide was fairly unstructured over the range of pH and temperatures examined, averaging 4% α-helix, 34% β-sheet, 16% β-turn, and 46% disordered structure (Table S3).

When cooled from 25.0 °C to 5.0 °C, the negative CD band at 196 nm of the peptide in pH 8 buffer decreased from −17,300 deg cm^2^ dmol^−1^ to −19,000 deg cm^2^ dmol^−1^ (Figure 8B). Curiously, a weak positive peak appeared at 228 nm, with an intensity of 140 deg cm^2^ dmol^−1^ at 15 °C and 550 deg cm^2^ dmol^−1^ at 5 °C.

No significant conformational changes were suggested by deconvolution analysis (Table S3), but the appearance of a single isosbestic point at ~188 nm in the pH curves suggested a shift between two conformational states. Difference spectra between the pH 8 and pH 5 data (Figure 8A, inset) showed a minimum at 196 nm, which is characteristic of an unfolded polypeptide [41]. Interestingly, the difference spectrum between the 25 °C and the 5 °C data showed a minimum at ~195 nm and a maximum at ~222 nm (Figure 8B, inset), suggesting that the carboxy-terminal domain also has PPII helical structure [38].

## 4. Discussion

Previous studies have shown that the aquaporin PvTIP3;1 from *P. vulgaris* seed is serine phosphorylated [7,9], which increases water channel activity [2]. To extend these studies, we sought to examine the overall pattern of membrane protein phosphorylation over the course of seed maturation and germination.

By the use of phosphoaminoacid-specific immunolabeling, we determined that serine and threonine phosphorylation decreases during seed maturation while levels of tyrosine phosphorylation remain relatively constant (Figure 1). Our studies also revealed that PvTIP3;1 is the primary membrane phosphoprotein, whereas PvTIP3;2 is expressed at very low levels (Figure 3 and Figure 4). Phosphorylation of Ser7 increases four-fold prior to seed dormancy (Figure 5), which may prime the PSV for water uptake upon seed imbibition. Unexpectedly, PvTIP3;2 becomes tyrosine phosphorylated following seed maturation (Figure 6), which may suggest a mechanism for the regulation of PvTIP3;2 following seed germination.

CD spectroscopy suggested that phosphorylation of Ser7 appears to induce some polyproline II helical structure in the amino-terminal domain of PvTIP3;1 (Figure 7). Analysis of the C-terminal domain showed that increasing pH increases polypeptide disorder (Figure 8).

### 4.1. The Membrane Phosphoproteome Changes Dynamically during Seed Maturation, Dormancy and Germination

Protein kinase and phosphatase activities vary dramatically during plant seed development and germination [14,42,43]. Generally, protein phosphorylation increases during the early and mid-maturation stages, decreases later in maturation and seed dormancy, then increases transiently in early germination. Concomitantly, protein phosphatase activity is highest after the initial stages of seed germination. In winged bean seed, protein kinase activity increases during development and peaks at the mid-maturation stage, then falls rapidly as the seed dries [44]. Kinase activity in this seed increases slightly upon imbibition, but then decreases during germination. In agreement with these previous results, we observed that levels of serine and threonine phosphorylated *P. vulgaris* seed membrane proteins decreased during seed maturation and after germination (Figure 1A,B, [7]); however, labeling of the few proteins showing tyrosine phosphorylation remained fairly constant (Figure 1C). In contrast, in Scots pine seed, extensive tyrosine phosphorylation occurs in a different temporal pattern, with a decrease in labeling of several proteins after germination, while others show an increase [45].

When we examined overall membrane protein abundance in *P. vulgaris* seed and compared this pattern with the overall membrane phosphoprotein content (Figure 2A,B), we found a correlation with a 24 kDa protein that we suspected was PvTIP3;1. This was indicated both by immunolabeling of a 24 kDa protein with antisera to full-length PvTIP3;1 (Figure 2C) and by mass spectrometry of the primary phosphoprotein (Figure 3). However, our MALDI-MS also detected two peptides of PvTIP3;2. Dual localization of TIP3;1 and TIP3;2 has been seen in *A. thaliana* [6] so this possibility is not surprising. However, PvTIP3;2 does not co-purify with PvTIP3;1 [9] and does not have detectable mRNA expression in seed [11]. Therefore, we infer that PvTIP3;2 is present only at very low levels. Consequently, PvTIP3;1 is the predominant phosphorylated membrane protein in dry and germinating seed. Previous studies have shown that PvTIP3;1 accumulates during seed development in the membranes of PSVs and attains its maximum level during the mid-maturation stage and then disappears several days after germination [46]. Vacuole membrane aquaporins are abundant in seed and are thought to mediate PSV rehydration and thereby aid germination [8]. Support for this function is suggested by the observation that treating *A. thaliana* seed with the aquaporin inhibitor mercury chloride delays the earliest stages of germination [12]. However, mercury has pleiotropic effects, and further characterization of the seed membrane phosphoproteome will be valuable for understanding seed maturation and germination.

### 4.2. Phosphorylation-Dependent Appearance of Polyproline II Secondary Structure

CD spectroscopy revealed that the amino terminus of PvTIP3;1 is largely disordered, which is expected for a domain bearing a phosphorylation site [47]. CD can detect α-helices that are seven to 10 amino acids, or two helical turns, in length [48]. The TIP3;1 amino and carboxy-terminal peptides did not show any α-helical propensity (Table S2), even though they are well over the minimum size. However, the weak positive CD band at 220 nm and the strongly negative CD band at ~196 nm (Figure 7B) indicates that the phosphorylated amino-terminus of PvTIP3;1 likely contains some level of PPII helicity [41]. This observation corresponds with findings that phosphorylation sites are enriched in PPII domains [49] and that serine phosphorylation can induce the formation of PPII helical structure [50]. That the maxima are blue-shifted in PvTIP3;1 compared to polyproline is likely due to the differences in transition energies between amino and imino peptide bonds [41]. In future experiments we hope to determine what this structural shift in PPII helix content could mean for phosphorylation-dependent channel opening in PvTIP3;1.

The carboxy-terminal domain of PvTIP3;1 has been implicated in aquaporin trafficking and regulation [3], and CD spectroscopy of the corresponding 23 amino-acid peptide showed both pH- and temperature-dependent shifts in the observed positive and negative maxima (Figure 8). Deconvolution suggested that the secondary structure is primarily comprised of a mix of β-strand and disordered regions (Table S3). At lower sample temperatures a weak CD maximum appeared at 228 nm, and the strong negative bands at 203 and 197 nm increased, which are characteristic features of a PPII helix [41]. A difference spectrum indicated an increase in disorder with an increase in alkalinity (Figure 8A). This may be caused by a histidine triplet within this domain (Figure S1) as an acidic environment would promote an ordered extended helical form driven by the mutual repulsion of protonated histidine residues. Additionally, the PvTIP3;1 carboxy-terminus is rich in proline, so an extended helical structure is possible, and we speculate that the presence of several histidine residues may enable pH-dependent tuning of this domain.

### 4.3. Serine Phosphorylation of PvTIP3;1 during Seed Maturation

The water channel activity of plant aquaporins may be regulated by pH, heteromerization, temperature, expression level and phosphorylation [51]. PvTIP3;1 was previously shown to be phosphorylated in vivo on Ser7 by a membrane-bound CDPK [7] and our results confirm this observation (Figure 3). When expressed in *X. laevis* toad oocytes, PvTIP3;1 showed a water permeability that was enhanced concomitantly with the activation of endogenous cAMP-dependent protein kinases [2]. These results indicate that the PvTIP3;1 water channel is gated by serine phosphorylation. Mutation of Ser7, Ser23, and/or Ser99 suggested that these three sites were all involved in phosphorylation-mediated channel gating [2]. However, only phosphorylation of Ser7 has ever been observed [7,9] (Figure 3), suggesting that Ser23 and Ser99 may not be accessible for phosphorylation in vivo or may affect channel function indirectly. Curiously, commercial phosphoserine antisera did not label phosphorylated PvTIP3;1 (Figure 5A), perhaps due to steric hindrance and/or electrostatic repulsion by neighboring amino acids. Therefore, to observe changes in phosphorylation during development, we generated polyclonal antibodies using Ser7 dephosphorylated and phosphorylated peptide antigens. The new TIPntS7P antiserum showed that phosphorylation occured following seed maturation, with a four-fold increase prior to the dry seed stage (Figure 5D). We therefore infer that the peak of water channel activity occurs in dry and germinating seed. The roughly four-fold increase in phosphorylation might imply that the number of Ser7-phosphorylated subunits in the PvTIP3;1 tetramer increases from one to four after seed maturation. However, MALDI MS indicated that Ser7 is only partially phosphorylated at the dry seed stage [9] and in seed imbibed for 24 h (Figure 3). This suggests either that not all PvTIP3;1 subunits are phosphorylated in the aquaporin homotetramer, or that only a subset of the PvTIP3;1 tetramers are fully phosphorylated, while others remain unphosphorylated or partially phosphorylated.

### 4.4. Role of PvTIP3;2 in Seed Development

TIP3;2 (β-TIP, [10]) was initially identified as a low-abundance protein in oil bodies from *A. thaliana* seed [52]. Both *AtTIP3;1* and *AtTIP3;2* transcripts show similar expression levels in seed [12], and a later study showed that TIP3;2 is co-localized with TIP3;1 in *A. thaliana* cotyledons [6]. Its substrate specificity will require some clarification, however. For instance, OsTIP3;2 from rice showed glycerol but not water transport activity in a *X. laevis* oocyte assay [53], while AtTIP3;2 appears to permit transmembrane H_2_O_2_ flux in an in vivo yeast assay system [54]. PvTIP3;2 lacks a serine residue in tandem with a consensus PKA site in its amino-terminal domain and is, therefore, not likely to be phosphorylated by the same mechanism as PvTIP3;1. Interestingly, the residue in PvTIP3;2 that is homologous to the phosphorylated Ser7 of PvTIP3;1 is the phosphomimetic amino acid glutamate, suggesting a constitutive response in this protein. Mass spectrometry of the gel-purified major phosphoprotein from *P. vulgaris* seed membranes shows only two mass peaks corresponding to peptides of TIP3;2 (Figure 3). Barring any substantial post-translational modifications, the two isoforms are very close in mass and pI, suggesting that PvTIP3;2 would likely have been co-purified and should have been detected in previous studies [9] if it were abundant. Consequently, we infer that PvTIP3;2 is a minor membrane protein in seed.

### 4.5. Possible Roles of PvTIP3;2 Tyrosine Phosphorylation

A number of plant aquaporins, including PvTIP3;1, are regulated by serine phosphorylation [3]. However, we are not aware of any data suggesting that water channel activity is directly regulated by tyrosine phosphorylation. Phosphoamino acid immunolabeling of seed membrane fractions showed that a 24 kD protein was tyrosine phosphorylated (Figure 1C). The pattern of labeling mimicked the abundance of the PvTIP3 proteins, and phosphotyrosine immunoprecipitation showed that PvTIP3;2 was tyrosine phosphorylated (Figure 6). Human aquaporin-1 (AQP1) is tyrosine phosphorylated on Y253 [55], but to our knowledge PvTIP3;2 is the first plant aquaporin demonstrated to undergo tyrosine phosphorylation.

Tyrosine phosphorylation of AQP1 was found to enable cGMP-regulated cation conductance of the pore in the center of the AQP1 tetramer [55], and a tyrosine kinase inhibitor was able to affect ion conductance of the MIP-family protein BIB expressed in *X. laevis* toad oocytes [56]. These results suggest that such phosphorylation could modulate alternative aquaporin protein functions. The appearance of tyrosine phosphorylated PvTIP3;2 long after the protein accumulation phase during seed maturation, but soon before protein disappearance following embryo germination, suggests a possible role in protein degradation.

Characterizing the site, role, and regulation of tyrosine phosphorylation in PvTIP3;2 will be an interesting target for further study. This post-translational modification is relatively unknown in plant seed development and germination, and may indicate a previously unknown regulatory mechanism in this stage of development.

### 4.6. A Model for the Role of PvTIP3;1 Ser7 Phosphorylation in Seed Development

Transcriptome analysis indicates that many TIP and plasma membrane intrinsic protein (PIP) genes are expressed in the developing seed of *P. vulgaris* [11,57], as well as in the seed of *A. thaliana* [12], rice [58] and barley [32], but little data are available regarding protein abundance. This is crucial for understanding the functional roles of these proteins since much of the aquaporin mRNA in seed appears not to be translated until later in development [6]. For instance, despite the variety of aquaporin mRNA present in *A. thaliana* seeds, only TIP3;1 and TIP3;2 show significant expression, whereas the PIP1, PIP2 and TIP1 aquaporins do not appear until after germination [12].

**Figure 9:**
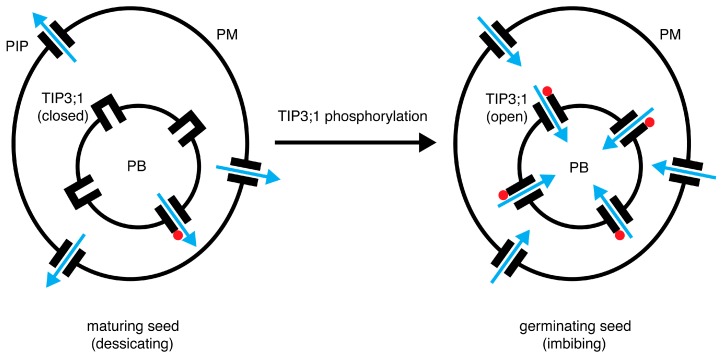
**A model** for the role of PvTIP3;1 Ser7 phosphorylation in seed development. Aquaporins may facilitate dessication during seed maturation. During seed germination, phosphorylated PvTIP3;1 facilitates the uptake of water into protein bodies. Proteolytic degradation of stored proteins supply the developing seedling with amino acids. Blue arrows show the direction of water flow, and red dots denote phosphorylation at Ser7. PIP, plasma membrane aquaporin; PB, protein body; PM, plasma membrane.

As a strict aquaporin, TIP3;1 is selective for water and impermeable to ions and small non-polar solutes [2,8]. The protein accumulates in PSV membranes during embryo maturation and disappears within several days after germination [5,54,59]. Treatment of seed with the aquaporin inhibitor mercury chloride will delay germination [12]. These results suggest that TIP3;1, and likely also its close homolog TIP3;2, are important for embryo maturation and/or seed germination [6,12,54,60]. Whether PvTIP3;1 and PvTIP3;2 function primarily during seed development or after germination is unclear. Interestingly, the PSVs of germinating pumpkin cotyledons are much less fluid than the tonoplasts of subsequently reformed vegetative vacuoles [61] and, hence, less permeable to water. These results suggest that the TIP3 proteins are the primary path for PSV water flux and that regulation of TIP3 aquaporins would control water movement into and out of the PSV (Figure 9). Although the mechanism is unknown, PIP and TIP aquaporins are linked to seed desiccation tolerance in maize [62], and the TIP3 aquaporins, in particular, are important in desiccation tolerance and longevity in *A. thaliana* seed [54]. Subsequently, water uptake in germinating pea seed appears to be facilitated by aquaporins since imbibition is slowed by the inhibitor 4-chloromercuribenzoic acid [63], and functional PIP aquaporins are found in the *P. vulgaris* seed coat [64], suggesting that in legume seeds both plasma membrane and the tonoplast aquaporins function to regulate water influx during germination.

## Figures and Tables

**Table 1:** Molecular weight and relative abundance of seed membrane proteins. Gel band staining ranked per lane in quartiles of intensity indicated as ++++ > +++ > ++ > + and not detected (-).

kD	Immature Seed	Mature Seed	Dry Seed	24 h Imbibed	48 h Imbibed
70	**+**	**++**	**++**	**++**	**+++**
67	**+++**	**++++**	**++**	**++**	**+++**
60	**-**	**+**	**++**	**+**	**++**
52	**++++**	**+++**	**++**	**+++**	**++++**
48	**++++**	**+++**	**++**	**+++**	**++++**
45	**+**	**+**	**++**	**++**	**+++**
36	**+**	**-**	**-**	**-**	**-**
33	**+**	**+**	**-**	**+**	**+**
32	**+**	**++**	**+**	**+**	**+**
29	**++**	**++**	**+**	**+**	**++**
24	**++++**	**++++**	**++++**	**++++**	**++++**
22	**++++**	**++++**	**+++**	**++++**	**++++**
19	**-**	**+**	**++**	**+**	**+**
15	**-**	**+**	**+**	**+**	**+**
12	**+**	**+**	**-**	**-**	**-**

**Table 2:** Molecular weight and relative abundance of seed membrane phosphoproteins. Gel band staining ranked per lane in quartiles of intensity indicated as ++++ > +++ > ++ > + and not detected (-).

kD	Immature Seed	Mature Seed	Dry Seed	24 h Imbibed	48 h Imbibed
24	**+**	**+**	**++++**	**++++**	**+++**
19	**-**	**-**	**+**	**-**	**-**

## Supplementary Materials

The following are available online at https://www.mdpi.com/2073-4409/8/11/1362/s1, Figure S1: Amino acid sequence alignment of PvTIP3;1 and PvTIP3;2, Figure S2: Validation of isoform-specific antisera by immunodetection of PvTIP3 proteins expressed in *Pichia pastoris*, Table S1: Relative signal intensity of phosphoaminoacid immunolabeled *P. vulgaris* seed membrane proteins, Table S2: Secondary structures of PvTIP3;1 amino-terminal peptides aTIPnt14 and Ser7-phosphorylated peptide aTIPnt14_pS7 by deconvolution of circular dichroism spectra, Table S3: Secondary structures of PvTIP3;1 carboxy-terminal peptide aTIPct23 by deconvolution of circular dichroism spectra.
