# Supplementary material for: Phosphorylation of TIP3 Aquaporins during Phaseolus vulgaris Embryo Development
Source: Cells. 2019 Oct 31;8(11):1362. doi: 10.3390/cells8111362 (PMC6912600; doi:10.3390/cells8111362)
Supplement: Supplementary file 1 [file cells-08-01362-s001.pdf]

cells MDPI

Table S1. Relative signal intensity of phosphoaminoacid immunolabeled *P. vulgaris* seed membrane proteins. Proteins immunolabeled with either (**A**) phosphoserine, (**B**) phosphothreonine, or (**C**) phosphotyrosine antisera, from Figure 1. Labeling ranked in quartiles of intensity such that ++++ > +++ > ++ > + and not detected (-). Blot lanes: IS, immature seed; MS, mature seed; DS, dry seed; 24, seed imbibed for 24 h; 48, seed imbibed for 48 h.

A	kD	immature seed	mature seed	dry seed	24h imbibed	48h imbibed
	53	+	+	+	-	-
	50	+	+++	+++	+	+
	46	+++	++++	-	-	-
	34	++	++	++	++	++
	30	++	++	+	+	+
	24	++	+	+	+	-
	20	++	+	-	-	-
	18	++	+	+	+	+
	12	++	++	++	+	++
	11	++	++	++	+	++
	8	++	+	+	+	+
	5	+	-	-	-	-
B						
	62	+	+	-	-	-
	52	++	+	+	+	+
	42	+	+	+	+	-
	34	-	++	+	+	-
	29	++	+	+	+	-
	27	+	+	+	+	-
	23	++	++	+	+	+
	19	+	+	-	-	-
	17	+	+	+	+	+
	14	+	-	-	-	-
	11	++++	+++	++	+	+
	7	++	++	-	-	-
	5	+++	+	+	-	-
C						
	24	++	+++	++++	++++	++++
	17	+	++	++	+	++
	11	+	++	+++	++	+
	9	+	++	++	-	++
	7	+	+	+	-	+

Table S2. Secondary structures of PvTIP3;1 amino-terminal peptides aTIPnt14 and Ser7-phosphorylated peptide aTIPnt14_pS7 by deconvolution of circular dichroism spectra. Deconvolution solutions produced by the CDSSTR method, using a reference dataset of 128 membrane and soluble protein structures [25]. Sample pH and temperature indicated. Secondary structure fractions noted are regular α-helix (α_R), distorted α-helix (α_D), regular β-strand (β_R), distorted β-strand (β_D), β-turn (T), and unordered (U), as defined by Sreerama *et al*. [65]. The normalized root mean square deviation (NRMSD) is shown for each solution.

peptide	pH	ºC	α_R	α_D	β_R	β_D	T	U	NRMSD
PvTIPnt14	5	25	0.00	0.02	0.23	0.14	0.12	0.49	0.025
PvTIPnt14	6	25	0.00	0.02	0.23	0.13	0.13	0.48	0.026
PvTIPnt14	7	25	0.00	0.02	0.23	0.14	0.11	0.48	0.029
PvTIPnt14	8	25	0.00	0.02	0.23	0.13	0.12	0.49	0.028
PvTIPnt14	8	15	0.00	0.01	0.23	0.14	0.11	0.50	0.022
PvTIPnt14	8	5	0.00	0.01	0.21	0.14	0.11	0.52	0.027
PvTIPnt14_pS7	5	25	0.00	0.01	0.23	0.14	0.12	0.50	0.021
PvTIPnt14_pS7	6	25	0.00	0.01	0.23	0.13	0.13	0.49	0.021
PvTIPnt14_pS7	7	25	0.00	0.01	0.22	0.13	0.13	0.50	0.023
PvTIPnt14_pS7	8	25	0.00	0.01	0.23	0.13	0.13	0.49	0.022
PvTIPnt14_pS7	8	15	0.00	0.00	0.22	0.13	0.12	0.52	0.027
PvTIPnt14_pS7	8	5	0.00	0.00	0.21	0.14	0.10	0.54	0.020

Table S3. Secondary structures of PvTIP3;1 carboxy-terminal peptide aTIPct23 by deconvolution of circular dichroism spectra. Deconvolution solutions produced by the CDSSTR method, using a reference dataset of 128 membrane and soluble protein structures [25]. Sample pH and temperature indicated. Secondary structure fractions noted are regular α-helix (α_R), distorted α-helix (α_D), regular β-strand (β_R), distorted β-strand (β_D), β-turn (T), and unordered (U), as defined by Sreerama *et al.* [65]. The normalized root mean square deviation (NRMSD) is shown for each solution.

pH	°C	α_R	α_D	β_R	β_D	T	U	NRMSD
5	25	0.00	0.03	0.21	0.12	0.17	0.45	0.013
6	25	0.01	0.03	0.22	0.12	0.17	0.45	0.013
7	25	0.01	0.04	0.22	0.13	0.16	0.44	0.015
8	25	0.00	0.05	0.21	0.12	0.14	0.47	0.018
8	15	0.01	0.02	0.22	0.12	0.15	0.47	0.015
8	5	0.00	0.03	0.20	0.12	0.15	0.49	0.012

```
PvTIP3;1     MA-TRRYSFGRTDEATHPDSMRASLAEFASTFIFVFAGEGSGLALVKIYQ 49
PvTIP3;2     MATTRRYEFGM-NEASHPDSMRATMAEFFSTCIFVFVGEGSVLALNHIYK 49
             ** ****.**  :**:*******::*** ** ****.**** *** :**:

PvTIP3;1     DSAFSAGELLALALAHAFALFAAVSASMHVSGGHVNPAVTFGALIGGRIS 99
PvTIP3;2     EPGSSATELVVVAVAHAFALFAAISISAHVSGGHVNPAVTFGALLGGRIS 99
             :.. ** **:.:*:*********:* * ****************:*****

PvTIP3;1     VIRAVYYWIAQLLGSIVAALVLRLVTNNMRPSGFHVSPGVGVGHMFILEV 149
PvTIP3;2     VLKALYYWIAQILGSIVAALLLRLVTNNMRPEGFGLSSGVGAVHGLILEI 149
             *::*:******:********:**********.** :*.***. * :***:

PvTIP3;1     VMTFGLMYTVYGTAIDPKRGAVSNIAPLAIGLIVGANILVGGPFDGACMN 199
PvTIP3;2     SLTFGLMYTVYATAIDPKRGAISTIAPLAIALVVGANILAGGPFDGACMN 199
              :*********.*********:*.******.*:******.**********

PvTIP3;1     PALAFGPSLVGWQWHQHWIFWVGPLLGAALAALVYEYAVIPIEPPPHHHQ 249
PvTIP3;2     PARAFGPALVGWRWQYHWIFWVGPLLGAALAAVLYEIVLVPIEPP--HHQ 247
             ** ****:****:*: ****************::** .::*****  ***

PvTIP3;1     PLATEDY 256
PvTIP3;2     PLPAEDY 254
             **.:***
```

Figure S1. Amino acid sequence alignment of PvTIP3;1 and PvTIP3;2 (from Genbank EST GW888595). The two proteins exhibit ~75% identity. Asterisks denote identical residues, double dots indicate conservative substitutions, single dots indicate semi-conservative substitutions. Protein sequences shown in single letter amino acid code. Sequences in boldface correspond to the amino-terminal acetylated peptides aTIPnt13C and bTIPnt15C used for generating antisera to PvTIP3;1 and PvTIP3;2, respectively. Peptides using the highlighted sequence in PvTIP3;1 were used for circular dichroism spectrometry studies.

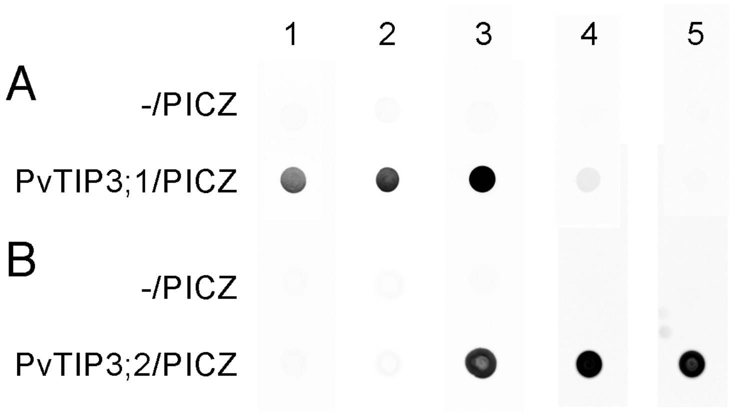

Figure S2. Validation of isoform-specific antisera by immunodetection of PvTIP3 proteins expressed in *Pichia pastoris*. (**A**) Dot blots of washed microsomes from either vector-only transformed *P. pastoris* (-/PICZ) or PvTIP3;1-expressing *P. pastoris* (PvTIP3;1/PICZ) spotted onto nitrocellulose filters. Filters then immunolabeled with antisera to aTIPnt13C (1), aTIPnt13C_S7P (2), full-length PvTIP3;1 (3), bTIPnt15C (4), or bTIPnt15C preincubated with peptide PvTIPnt14 (5). (**B**) Immunodetection of PvTIP3;2 expressed in *P. pastoris*. Dot blots of washed microsomes from either vector-only transformed *P. pastoris* (-/PICZ) or PvTIP3;2-expressing *P. pastoris* (PvTIP3;2/PICZ) spotted onto nitrocellulose filters. Filters immunolabeled with same antisera as in **A**.
